# Design of plasmonic nanomaterials for diagnostic spectrometry

**DOI:** 10.1039/c8na00319j

**Published:** 2018-11-23

**Authors:** Deepanjali Dattatray Gurav, Yi (Alec) Jia, Jian Ye, Kun Qian

**Affiliations:** School of Biomedical Engineering, Med-X Research Institute, Shanghai Jiao Tong University Shanghai 200030 People's Republic of China k.qian@sjtu.edu.cn yejian78@sjtu.edu.cn; School of Environment and Science, Queensland Micro- and Nanotechnology Centre, Griffith University, Nathan Campus Queensland 4111 Australia

## Abstract

Molecular diagnostics relies on the efficient extraction of biomarker information from the given bio-systems. Plasmonic nanomaterials with tailored structural parameters are promising for the development of biomarker assays due to enrichment effect and signal enhancement. Herein, we overview the recent progress on the development of plasmonic nanomaterials for diagnostic spectrometry, encompassing the interface, mechanism, and application of these materials. For interface, we summarized the types of plasmonic nanomaterials used as interfaces between different materials and light. For mechanism, we descirbe the key parameters (*e.g.*, hot carriers and heat) that characterize the plasmonic effect of materials. For application, we highlighted recent advances in matrix assisted laser desorption/ionization mass spectrometry (MALDI MS) and surface enhanced Raman spectroscopy (SERS) toward precision in *in vitro* and *in vivo* diagnostics. We foresee the upcoming era of precision diagnostics by nano-assisted spectrometry methods in both academy and industry, which will require the interest and effort of scientists with diverse backgrounds.

## Abstract 2

Development of plasmonics for diagnostic spectrometry encompassing the interface, mechanism, and application of nanomaterials.

## Graphical abstract

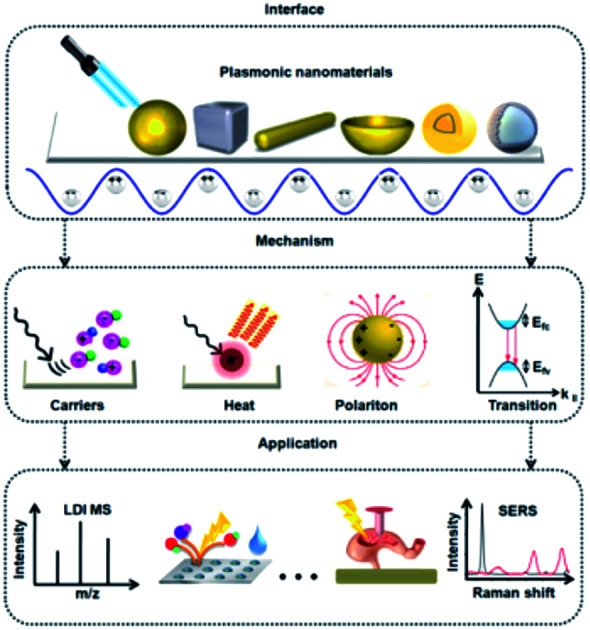

## 1. Introduction

Diagnostics is crucial in biomedical science and clinical practice with the aim of better healthcare worldwide.^1,2^ Particularly, molecular diagnostics relies on the efficient extraction of biomarker information from bio-systems, such as bio-fluids and tissues.^3–5^ The detection of biomarkers in bio-fluids (*e.g.*, serum) affords low invasiveness and high adaptability towards point-of-care (POC) applications.^6,7^ In parallel, the detection of biomarkers in tissues serves as the gold standard for precision diagnosis in pathological examination.^5,8^ However, it is difficult to detect biomarkers in real cases owing to the high complexity of biological samples and low abundance of target molecules.^9,10^ Currently, nanomaterials with tailored structural characteristics exhibit superior enrichment effects and signal enhancement in molecular diagnostics, which significantly boost the development of biomarker assays.

Engineered plasmonic nanomaterials have defined chemo-physical properties, and used in a series of state-of-the-art applications with unique mechanisms.^5,10,11^ Typically, the design of plasmonic nanomaterials requires consideration of composition, morphology, and surface chemistry.^12,13^ With regard to composition, noble metals (*e.g.*, Au and Ag) are the most used candidates for desirable surface plasmons.^6,14,15^ With regard to morphology, micro/nanostructures may provide distinct light responses for personalized use.^16,17^ With regard to surface chemistry, specific molecular probes on the surface afford efficient molecular capture and recognition.^13,18^ Moreover, engineered plasmonic nanomaterials should be integrated with a device for assays.^6,19,20^ To date, advanced spectrometric methods require the rational design of plasmonic nanomaterials that address all the above considerations, which require interdisciplinary research efforts towards application in the diagnostics field.

Spectrometry methods display advantages in terms of sensitivity, throughput, and cost compared with conventional diagnostic methods such as physical examination.^10^ Among the diagnostic spectrometry methods, MALDI MS^14,21^ and SERS^22,23^ are two prominent emerging tools. In MALDI MS, matrix materials determine the LDI efficiency with identification of diverse molecules over a broad mass range.^6,14,24^ In SERS, substrate materials enhance the Raman signals for ultra-sensitive detection even at the single molecule level.^23,25^ Notably, both MALDI MS and SERS require engineered nanomaterials with tuneable plasmonic properties, considering the key role of the interface between materials and light in both methods.

**Fig. 1:**
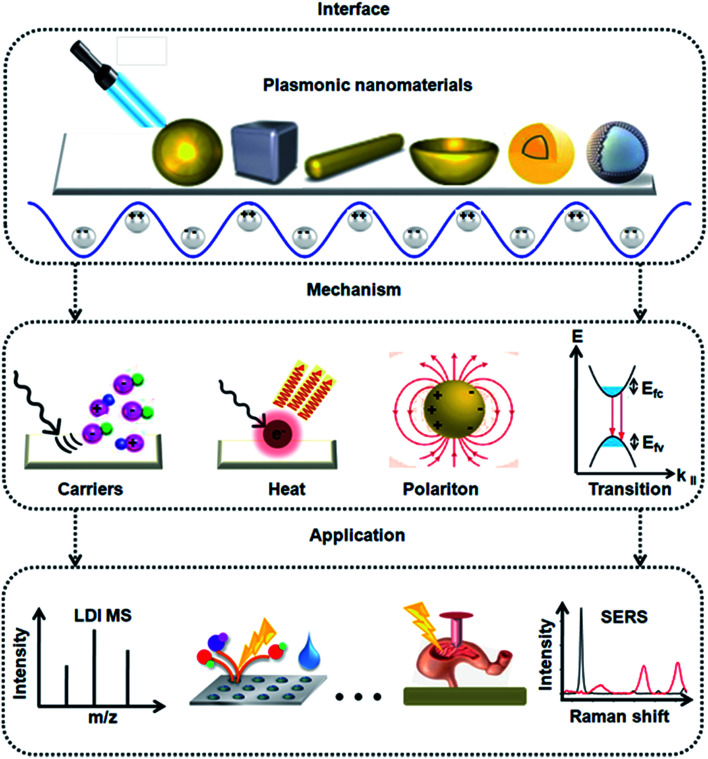
Schematics of the development of plasmonic nanomaterials for diagnostic spectrometry. We summarize the types of plasmonic nanomaterials, addressing the interface between different materials and light. The mechanism demonstrating key parameters such a generation of hot carriers, heat, surface plasmon polariton, and interband transitions to characterize the plasmonic effect of these materials. With regard to applications, we highlight the recent advances in MALDI MS and SERS for precision *in vitro* and *in vivo* diagnostics.

In this review, we discuss the development of plasmonic nanomaterials for diagnostic spectrometry, as shown in Fig. 1, focusing on the interface, mechanism, and application of these materials. With regard to interface, we summarize the types of plasmonic nanomaterials, addressing the interface between the different materials and light. With regard to mechanism, we discuss the key parameters (*e.g.*, hot carriers and heat) that characterize the plasmonic effect of materials. With regard to applications, we highlight the recent advances in MALDI MS and SERS toward precision *in vitro* and *in vivo* diagnostics. We foresee the upcoming era of precision diagnostics with nano-assisted spectrometry methods applied in both academy and industry, which requires the interest and effort of scientists with diverse backgrounds.

## 2. Types of plasmonic nanomaterials

The unique opto-electronic properties of plasmonic nanomaterials impart characteristic features based on their size, shape, and composition.^13,26,27^ The controllable size and shape of these particles can be tailored by optimization of synthetic conditions, such as stabilizing agents,^28,29^ surface functionalization^15,30^ and temperature variations.^24,29^ Plasmonic nanomaterials are most commonly synthesized using chemical methods such as citrate/borohydride/ascorbic acid reduction,^14,30,31^ hydrothermal reactions,^32^ light-mediated processes, laser/γ-ray/electron beam irradiation, and lithography. Nanomaterials prepared through physical methods involve the deposition of thin films by condensing atomized elements or components by vapour evaporation or sputtering.^6^ The optical properties can be further modulated using other inorganic or organic entities including silica, carbon, polymer, and alloy materials. Thus, rationally designed nanostructures have enabled researchers to develop a series of diagnostic devices with high sensitivity and selectivity for biomedical applications.

**Fig. 2:**
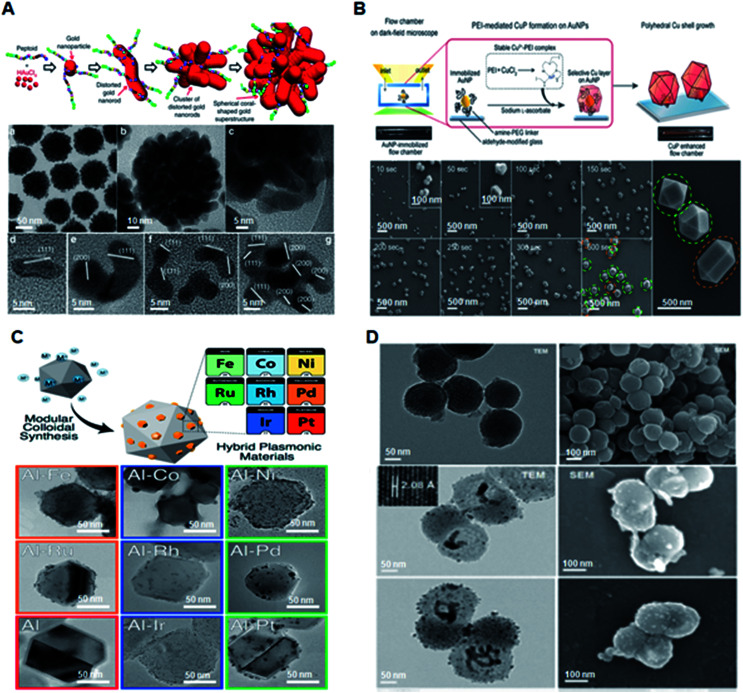
Engineered plasmonic nanomaterials for diagnostic applications. (A) Schematic of Pep-1-induced formation of spherical coral-shaped gold nanoparticles and TEM images demonstrating the monodispersity of the nanoparticles. Reproduced with permission.^30^ Copyright 2018, Nature Publishing Group. (B) Schematic of polyethyleneimine (PEI)-mediated overgrowth of Cu nanopolyhedra by highly specific CuP growth on AuNPs on a glass slide along with structural validation using scanning electron microscopy (SEM). Reproduced with permission.^38^ Copyright 2017, Wiley-VCH. (C) Synthesis of eight varieties of size-tunable transition-metal-decorated aluminum nanocrystals. Adapted with permission.^39^ Copyright 2017, American Chemical Society. (D) Schematic illustration and material characterization of Janus hybrids for detection of small metabolites. Reproduced with permission.^10^ Copyright 2018, Royal Society of Chemistry.

Plasmonic nanoparticles have evolved over the years from single metal nanoparticles to engineered hybrid structures such as alloys, and core–shell/nanoshell materials.^33,34^ Notably, Au and Ag nanoparticles have been extensively explored for diagnostics owing to their enhanced surface plasmon properties, tuneable structural parameters, and biocompatibility.^35–37^ Yan *et al.* developed a biomimetic approach for the controllable synthesis of Au nanoparticles with manipulated structural morphologies using sequence designed peptoid molecules (Fig. 2A).^30^ The engineered peptoid sequences predictively enabled the morphological evolution of Au nanoparticles from spherical to coral-like, thus exhibiting a plasmonic enhancement as high as 10^5^-fold. Nam *et al.* reported a facile method for the synthesis of polyhedral Cu nanoshells using Au as the core and Cu as the shell (Fig. 2B).^38^ The highly intensive and quantifiable optical signals provide a promising approach for quantitative onsite naked-eye detection. An alternative to traditional gold and silver nanoparticles was reported by Halas *et al.* and Ringe *et al.* (Fig. 2C).^39^ These groups reported a simple polyol synthesis route to produce eight varieties of transition metal-decorated aluminium nanocrystals as a promising material platform for plasmonic photocatalysis, surface-enhanced spectroscopy, and quantum plasmonics. Recently, Liu and Qian reported the application of plasmonic Janus hybrids as bio-analytical platforms (Fig. 2D).^10^ The proposed Janus hybrids were composed of periodic mesoporous organosilica (PMO) in conjunction with carbon spheres as core materials and were further loaded with Ag nanoparticles as nanoshells. Thus, the transition of plasmonic nanomaterials from the 1-dimensional to 2-dimensional form may provide a wide range of applications in bio-diagnostic research.

## 3. Mechanism of plasmonic nanomaterials

**Fig. 3:**
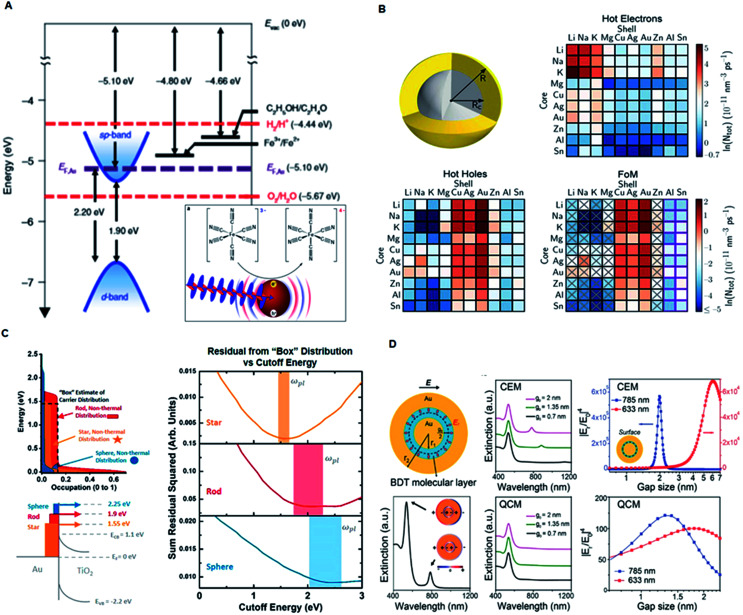
Mechanism of plasmonic nanomaterials. (A) Experimental model system demonstrating the electron transfer from plasmonically excited gold NPs. Adapted with permission.^36^ Copyright 2018, Macmillan Publishers Limited, Springer Nature. (B) Schematic illustration of a bimetallic core–shell nanoparticle for generation of hot electrons or hot holes with 100 different combinations. Reproduced with permission.^45^ Copyright 2018, Springer Nature. (C) Simplified hot-carrier model showing comparison of the injected hot-electron distributions in the semiconductor with a “box” distribution ranging from the Fermi level to the plasmon frequency. Reproduced with permission.^46^ Copyright 2018, American Chemical Society. (D) Gap-enhanced Raman tags (GERTs) for studying electron transport across plasmonic molecular nanogaps using the classical electromagnetic model (CEM) and quantum-corrected model (QCM). Adapted with permission.^35^ Copyright 2017, American Chemical Society.

Plasmonic nanomaterials typically show optical resonances due to the collective oscillation of excited quasi-free electrons, which leads to the formation of a dipole moment after coupling with electromagnetic radiation.^34,40,41^ These quasi-free electrons or “plasmons” generally localize and modify light at the metal surface, giving rise to two main types of surface plasmon resonance: (1) propagating surface plasmon polariton/resonance (SPP/SPR) along the metal–dielectric interface, and (2) localized surface plasmon resonance (LSPR) in the localized nanoscale region of metallic nanoparticles. These resonances are produced due to electron oscillations at particular frequencies dependent primarily on the shape, size, dielectric properties and composition of the nanostructures (Fig. 3A).^36^ Generally, LSPRs only occur in structures smaller than the wavelength of the excitation light. However, SPPs only require the presence of an interface between metal and air and may extend over several wavelengths. Label-free LSPR or SPP biosensors mainly rely on the resonance shift induced by the analytes binding to the metal surface due to the refractive index change around the plasmonic nanomaterials,^11,42,43^ which can reach an ultrahigh sensitivity down to the picomolar range.^16,38,44^

Plasmonic nanomaterials are also favorable for plasmon-enhanced laser desorption/ionization mass spectrometry (LDI MS) mainly due to two mechanisms: (1) amplified absorption cross section and (2) increased generation of hot carriers at the resonance wavelength.^6,14^ Larger absorption cross section induces greater photothermal effect, which results in a more efficient energy transfer to the analytes. The added energy results in a phase transition of the analytes from the condensed phase to the gas phase, ultimately generating gas phase analyte ions within the mass detector. The generation of hot carriers can also promote the photo-decomposition of complexes of metal ions and small metabolites (Fig. 3B and C).^45,46^ The emission wavelength of the irradiation source (laser) used in mass spectroscopy can be in the UV-NIR range. Therefore, Au^36^ and Cu^38^ nanoparticles (*e.g.*, nanospheres, nanoshells, and nanorods) are more suitable for the visible and NIR laser, and Ag nanoparticles are more suitable for the UV laser due to the different frequencies where interband transitions occur.^14,24,47^ Recently, we demonstrated that the anti-bonding modes of Au nanoshells can generate hot carriers in the UV range and consequently display superior sensitivity over traditional organic matrices for enhanced LDI MS-based detection and profiling of small metabolites in serum samples.^48^

SERS is another powerful spectroscopic technique providing ultrasensitive fingerprinting of target molecules up to the single molecule level. Au and Ag nanomaterial substrates are reported as sensors for SERS owing to their enhanced LSPR in the visible and near infrared regions.^18,19^ The SERS effect boosts the Raman signals of adsorbates on the surfaces of plasmonic nanostructures when their plasmon resonances and consequently the enhanced near field are excited at the wavelength of the stimulating laser beam (Fig. 3D).^35^ In general, SERS processes are based on the following well established mechanisms. The first mechanism is electromagnetic enhancement arising due to excitation of LSPRs when the incident light strikes the surface of the nanoparticle. The highest field enhancement is achieved when the plasmon frequency (*ω*_p_) is in resonance with the radiation and the plasmon oscillations are perpendicular to the surface. These requirements are fulfilled by plasmonic materials exhibiting rough surfaces and achieving maximum localized collective oscillation. The second mechanism is the chemical enhancement that arises due to the formation of charge transfer complexes between the chemically adsorbed species and the metal surface. This mechanism is prominent in analyte molecules that can directly bond to the surface of the substrate and exclude surface plasmons. Thus, when the analyte molecule is excited by visible light, an electronic transition occurs between the molecular orbitals of the adsorbate molecule, giving rise to the characteristic intensity signal. Overall, the formation of first and second-generation hotspots using engineered plasmonic nanomaterials greatly affects the overall SERS intensity for the sensitive detection of analytes at low concentrations.^49^

## 3. MALDI MS and SERS based diagnostics

Plasmonic nanomaterials have been widely explored for biological and chemical sensing applications owing to their unique surface plasmon properties.^50,51^*In vitro* diagnostic applications are mainly focused on the sensitive and accurate detection of biomolecules such as nucleic acids (DNA/RNA/siRNA/micro-RNA/plasmids), proteins, peptides, lipids and low molecular weight compounds.^52,53^ A plasmonic biosensor composed of gold nanohole arrays (Au-NHAs) was developed for the detection of biomarkers in the pg mL^−1^ range.^16^ Another study emphasized the ultrasensitive detection of telomerase activity in gastric and breast cancer tissues using Au nanowire SERS sensors.^23^ Plasmonic material-based *in vivo* techniques were successfully applied to the photothermal imaging of cells. Plasmonic nanobubbles were reported for intraoperative diagnostics and elimination of *in vivo* residual microtumors in head and neck cancer cells.^5^ Thus, tuning the SPR properties of plasmonic nanomaterials for biomolecular recognition can be beneficial for providing better alternatives for early disease diagnostics.

**Fig. 4:**
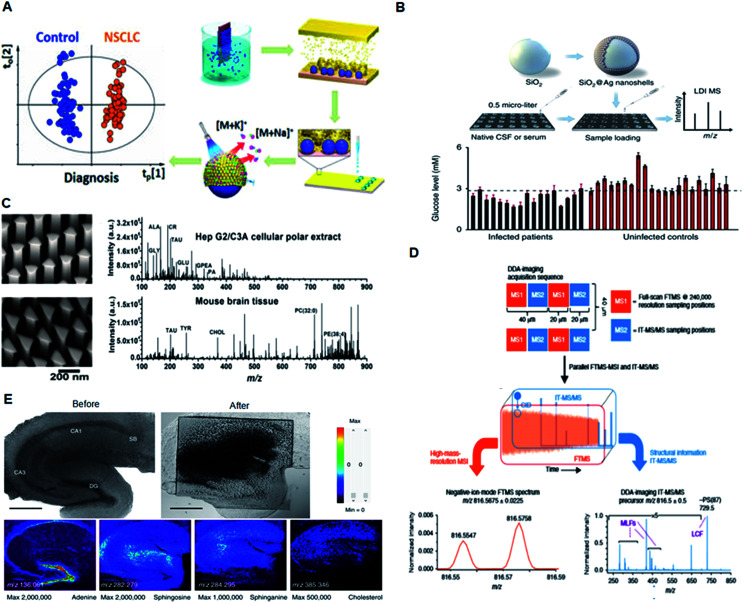
Application of plasmonic nanomaterials for LDI MS-based diagnostics. (A) MS detection of small molecules in serum and exosomes using plasmonic Au chips for diagnosis of early-stage lung cancer. Reproduced with permission.^6^ Copyright 2018, American Chemical Society. (B) LDI MS-based *in vitro* diagnostics of small metabolites using plasmonic silver nanoshells. Adapted with permission.^14^ Copyright 2017, Nature Publishing Group. (C) LDI MS of metabolites extracted from HepG2/C3A cells and mouse brain sections directly analyzed in positive ion mode using elevated bow tie nanostructures. Adapted with permission.^21^ Copyright 2018, Wiley-VCH. (D) Experimental workflow for data-dependent acquisition imaging and automatic structural identification of detected lipids using ALEX123 software. Adapted with permission.^8^ Copyright 2018, Springer Nature. (E) Optical images and mass spectrometric (MS) images of a mouse hippocampal tissue slice. Adapted with permission.^55^ Copyright 2017, Springer Nature.

A variety of biosensor platforms based on plasmonic nanomaterials with enhancements such as sensor platforms, molecular detection, and cell receptor analysis show promising outlooks from clinical perspectives.^54^ Qian *et al.* reported two independent studies based on a MALDI MS approach using plasmonic nanoshells for *in vitro* diagnostics of early-stage lung cancer patients and individuals with post-operative brain infections. A clear separation between the healthy and patient groups was achieved by the metabolic fingerprinting of biofluids, such as serum, exosomes and cerebrospinal fluid (Fig. 4A and B).^6,14^ Compared with the conventionally shaped plasmonic nanomaterials, Stopka *et al.* reported elevated bowtie shaped silicon nanopost arrays (NAPA) as a nanophotonic ionization platform for LDI MS (Fig. 4C).^21^ The deposition of triangular chromium on the silicon post pairs increased the ionization efficiency of the NAPA matrix by 17-fold, and the platform was utilized for trace analysis of biomolecules in complex biological samples. Thus, modulating the dimensions of a nanomaterial can provide efficient near-field enhancement of the electric field with improved resonance surface plasmons for the sensitive detection of analytes.

Apart from *in vitro* diagnostics, plasmonic nanomaterials have been applied as a multifunctional platform for MS-based *in vivo* studies. Recently, the Ejsing and Heeren proposed an automated MS imaging methodology for *in situ* structural identification of lipids (Fig. 4D).^8^ The method takes advantage of a newly developed matrix assisted laser desorption/ionization mass spectrometry imaging (MALDI-MSI) source and a hybrid ion trap-Orbitrap instrument. A single experiment resulted in a high-resolution MSI dataset with accurate mass measurements of all detected peaks and structural information for the nominal mass corresponding to all the lipids present in the cerebellar tissue extracts. Kim *et al.* reported a high spatial resolution MS system for imaging live hippocampal tissue slices under open-air atmospheric pressure (AP) and ambient temperature conditions at the subcellular level (Fig. 4E).^55^ MS imaging at a spatial resolution of 2.9 μm clearly revealed the differences between the molecular composition of the apical and basal dendrite regions of hippocampal tissue. Thus, MALDI MS approaches provide rapid, sensitive, accurate, and cost effective methods for the analysis and structural identification of low-abundance metabolites for clinical applications.

**Fig. 5:**
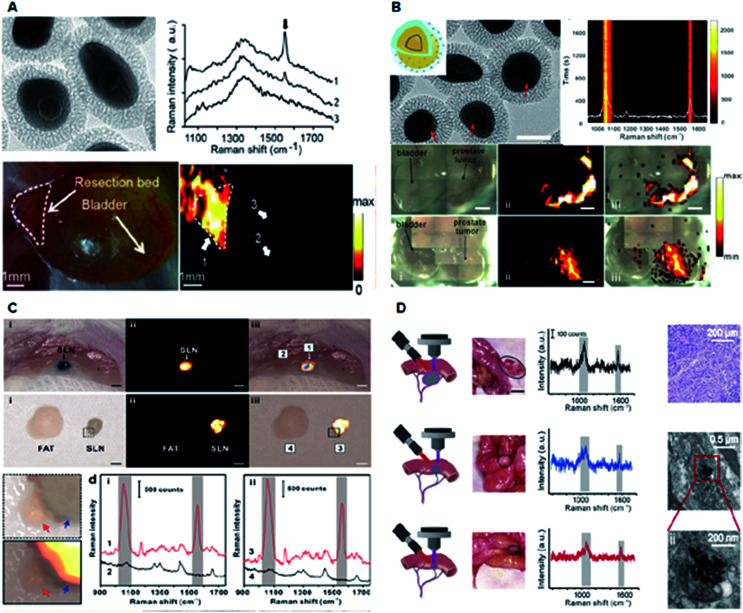
Application of plasmonic nanomaterials for SERS-based diagnostics. (A) Intraoperative Raman imaging of residual microtumors after surgical resection of primary tumors using gap-enhanced Raman tags (GERTs). Adapted with permission.^19^ Copyright 2018, American Chemical Society. (B) Ultraphotostable mesoporous silica-coated GERTs for high-speed bioimaging with a representative TEM image of GERTs, photostability measurement of time-resolved SERS spectra of solid MS GERTs on a silicon wafer during continuous irradiation for 30 min and imaging of orthotopic prostate tumors in mice in bright-field, and corresponding Raman images (2.21 × 10^5^ W cm^−2^ power density). Reproduced with permission.^58^ Copyright 2017, American Chemical Society. (C) High-contrast intraoperative Raman imaging of sentinel lymph nodes (SLNs). Adapted with permission.^22^ Copyright 2018, Elsevier B. V. (D) *In vivo* intraoperative Raman-guided chemo-photothermal therapy of advanced ovarian cancers with disseminated microtumors represented by schematic diagrams, disseminated tumors (indicated by the gray circles), the corresponding detected Raman signals, representative histological analyses of the microtumors obtained from mice, and the representative TEM images showing the presence of GERTs within the tumor cell. Adapted with permission.^61^ Copyright 2018, Wiley-VCH.

SERS nanoparticles can be utilized as optical labelling nanoprobes, similar to fluorescent dyes and quantum dots, for bioimaging and biosensing with the additional advantages of fingerprint vibrational signals as a unique optical code and ultra-narrow line widths for multiplexing.^22,56,57^ Typically, SERS efficiencies reach maximal values at resonant conditions, *i.e.*, when the excitation wavelength overlaps with the LSPR wavelength, preferably in the near-infrared (NIR) biological window. However, on-resonant photogenerated heat may inevitably disturb or even destroy a biological sample or induce photo-bleaching of the SERS signals during the imaging process. Recently, Ye *et al.* reported a new type of off-resonant SERS nanoparticles, termed gap-enhanced Raman tags (GERTs), for intraoperative cancer imaging (Fig. 5A and B).^19,58^ GERTs are composed of a uniform subnanometer-sized interior gap between the metallic core and shell. GERTs have also demonstrated a number of important optical properties, including the quantum plasmon effect,^35,59^ large Raman enhancement and highly tuneable multiplexed encoding capability,^12^ refractive index quantification of sub-nanometer-thick molecular layers on nanoparticles,^60^ ultrahigh photostability during continuous laser irradiation with an off-resonance strategy,^25,58^ and high-speed SERS cell imaging.^58^

GERTs have also been employed in sentinel lymph node (SLN) biopsy for investigating the lymphatic metastasis of malignant tumors. A quantitative volumetric Raman imaging (qVRI) data-processing method can be employed to acquire a high-resolution 3-dimensional (3D) margin of SLN as well as content variation of GERTs in the SLN (Fig. 5C).^22^ In addition, SLN detection could be realized *via* a cost-effective, commercial, and portable Raman scanner.^22^ Therefore, GERTs have great potential to be translated in clinical application for accurate and intraoperative location of the SLN. Recently, a crucial example of Raman imaging with GERTs is a robust platform developed for intraoperative detection and eradication of residual microscopic foci, which present problems in surgical margins, tumor invasion, and multifocal tumor spread.^19^ In an orthotopic prostate metastasis tumor model, the systematic delivery of GERTs enabled precise imaging and real-time ablation of macroscopic malignant lesions around a surgical bed without damaging normal tissues.^19^ Consequently, the GERTs-based surgery prevented local recurrence and delivered 100% tumor-free survival.

Another study reported a specifically designed cisplatin-loaded GERTs platform for the intraoperative detection and elimination of unresectable ovarian tumors (Fig. 5D).^61^ With unique and strong Raman signals, good biocompatibility, decent plasmonic photothermal conversion, and good drug loading capacity, GERTs enabled the detection and specific elimination of microtumors with a minimum diameter of 1 mm *via* chemo-photothermal synergistic therapy, causing minimal side effects and significantly prolonged survival in mice. Overall, the artificial regulation of the shape, size, shell thickness, and hybridization of plasmonic materials broadens their applications for *in vitro* and *in vivo* diagnostics.

Overall, diagnostic spectrometry methods, particularly MALDI MS, can be advantageous in terms of high throughput, sensitivity, selectivity, and accuracy without tedious sample preparation methods.^14,21,47^ Recent reports highlight the importance of engineered materials for the sensitive detection of analytes in real samples.^6,14,21,51,55^ Since the traditional bare metal nanoparticles as matrices displayed unsatisfactory performance, hybrid plasmonic nanomaterials would be better candidates due to their unique features, such as nanoscale surface roughness and high production of hot carriers.^45,46,48^ Hence, new approaches using novel hybrid plasmonic nanomaterials hold key importance for diagnostic applications dealing with real samples.

## 4. Other diagnostic spectrometry

Plasmonic nanomaterials have also been widely explored for use in diagnostic devices based on colorimetry, fluorescence, *etc.*^11,16^ Due to their fascinating optical properties, plasmonic nanomaterials have achieved major breakthroughs as colorimetric probes for the sensitive and selective detection of various biomolecules.^27^ Binding of a target molecule to plasmonic nanoparticles directly results in a color change that can be observed by shifting and broadening the LSPR peaks.^31^ As fluorescence sensors, plasmonic nanostructures can modify the incident electromagnetic field and increase the field amplitude, leading to an increase in the radiative scattering efficiency of a fluorophore.^37^ The emerging field of fluorescence spectroscopy known as near-infrared fluorescence (NIRF) offers high detection sensitivity and specificity in the range of 650 to 1700 nm.^62^ NIRF-based techniques exhibit lower background signals arising from biological targets compared with the UV-visible absorption-based techniques. Thus, the use of nanoengineered plasmonic materials affords distinct optical properties owing to their tunable structural parameters, long-term stability and enhanced detection ability.

**Fig. 6:**
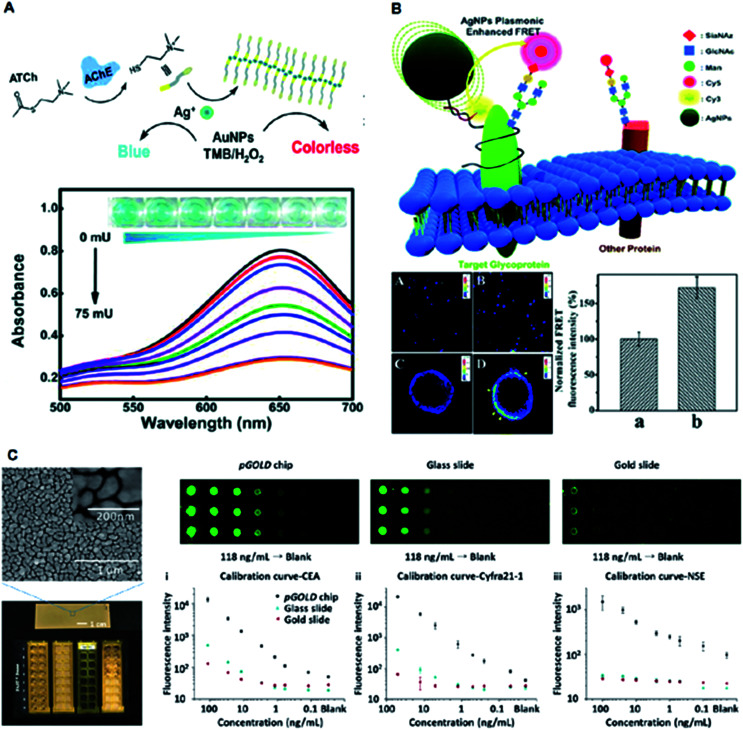
Application of plasmonic nanomaterials for other spectrometry-based diagnostics. (A) Colorimetric detection of acetylcholinesterase (AChE) activity with a schematic illustration of the sensing approach, UV-vis spectra of CTAB-AuNPs in the presence of various AChE concentrations, and the corresponding optical images of the mixtures. Adapted with permission.^31^ Copyright 2018, Wiley-VCH. (B) Schematic illustration of AgNPs-enhanced FRET imaging for protein-specific sialylation of the cell surface, with confocal images of protein-specific sialylation on the cell surface in the absence (A, C) and presence (B and D) of AgNPs, and quantitative analysis of the FRET signal intensity in the absence (a) and presence (b) of AgNPs. Adapted with permission.^37^ Copyright 2017, Royal Society of Chemistry. (C) Near-infrared fluorescence-enhanced biomarker detection using plasmonic gold chips probed by IRDye800, demonstrating the nanoscopic gold island morphology using SEM, and comparative fluorescence mapping results for different concentrations of analytes using the plasmonic gold chip (left), glass slide (middle), and gold slide (right) along with standard calibration curves. Adapted with permission.^62^ Copyright 2016, Wiley-VCH.

Typically, plasmonic optical sensors have gained popularity owing to their simplified synthesis methods and facile surface modifications. Zhang *et al.* reported a sensitive colorimetric method for the detection of acetylcholinesterase activity using cetyltrimethylammonium bromide-capped gold nanoparticles (CTAB-AuNPs) (Fig. 6A).^31^ The presence of Ag^+^ ions along with CTAB-AuNPs displayed enhanced peroxidase activity compared with similarly sized AuNPs. The metal-induced fluorescent enhancement has been translated for various biomedical applications such as bio-imaging and ultrasensitive immunoassays. Liu *et al.* utilized the Förster resonance energy transfer (FRET) property of Ag nanoparticles for direct imaging of protein-specific sialylation on the cell surface (Fig. 6B).^37^ Liu *et al.* constructed a plasmonic gold (pGOLD) substrate as a promising platform for NIRF detection of biological molecules in the range of ∼650–1700 nm (Fig. 6C).^62^ The nanostructured gold islands on pGOLD chips afforded multiplexed detection of a panel of biomarkers in lung cancer cells with high throughput, sensitivity of ∼52% and specificity of ∼96%. Overall, the use of nanomaterials in a variety of spectrometric techniques facile and ultra-sensitive diagnostics demonstrates their significant potential for clinical applications.

## Conclusion and prospects

In summary, we reviewed the typical cases of spectrometric diagnostics that use plasmonic nanomaterials with tailored design. Notably, non-metallic materials such as carbon^63,64^ and silicon^17,65^ with specific optical properties may be considered plasmonic, according to the broad definition of plasmonic nanomaterials.^50,64^ There have been series of bio-analytical applications based on non-metallic materials, similar to metallic plasmonic nanomaterials.^20,66^ These non-metallic materials would be promising alternatives to noble metals, mainly due to their comparable performance and lower costs. We anticipate three major research lines following this review from the perspective of these materials. On-going research will be focused on (1) novel materials with tailored composition, morphology, and surface chemistry; (2) new mechanisms with defined parameters to characterize the plasmonic effect; and (3) advanced devices with tunable performance based on the materials and plasmonics.

In parallel, from the perspective of applications, it would be desirable to emphasize: (1) multi-functional uses, *i.e.*, tackling several technical problems using one material; (2) combinational uses that offer a perfect solution using a combination of spectrometry methods; and (3) practical uses that may satisfy the urgent diagnostic needs in clinics and daily life. Therefore, we hope that this review would contribute to the better improvements in healthcare in future, ranging from fundamental research to commercialization.

## Conflicts of interest

There are no conflicts to declare.

## Supplementary Material
